# Amygdalin prevents *multidrug-resistant Staphylococcus aureus*-induced lung epithelial cell injury by regulating inflammation and oxidative stress

**DOI:** 10.1371/journal.pone.0310253

**Published:** 2024-09-16

**Authors:** Zhaolei Wang, Haixia Du, Haofang Wan, Jiehong Yang, Haitong Wan

**Affiliations:** 1 School of Life Sciences, Zhejiang Chinese Medical University, Hangzhou, Zhejiang, PR China; 2 School of Basic Medicine, Zhejiang Chinese Medical University, Hangzhou, Zhejiang, PR China; 3 Academy of Chinese Medical Sciences, Hangzhou, Zhejiang, PR China; Cardiff’s Metropolitan University: Cardiff Metropolitan University, UNITED KINGDOM OF GREAT BRITAIN AND NORTHERN IRELAND

## Abstract

*Methicillin-resistant Staphylococcus aureus* (MRSA) is an opportunistic pathogen that can cause severe bacterial pneumonia. Amygdalin is the main active pharmaceutical ingredient of bitter almond, which has broad-spectrum antibacterial, anti-inflammatory, anti-oxidation and immunomodulatory effects. It is also the main ingredient of Yinhua Pinggan granule, which is commonly used to moisten the lung and relieve cough. However, little is known about the effects of amygdalin on MRSA. In this study, we found that amygdalin exhibited good antimicrobial activity in *vitro* against MRSA. Amygdalin has a protective effect on MRSA infected cells, and the effect is better when combined with levofloxacin. It also can reduce the adhesion and invasion of MRSA to cells. Amygdalin has anti-inflammatory and antioxidant effects, which can significantly reduce the increase of inflammatory factors and the production of ROS caused by infection. The protective mechanism of amygdalin on cells may be related to inhibiting the expression of NLRP3, ASC and IL-1β pyroptosis pathways. Taken together, our study suggests that amygdalin exerts antibacterial effects by affecting biofilm formation, the expression of virulence factors, and drug resistance genes. Amygdalin combined with levofloxacin has a protective effect on A549 cells infected with MRSA, and the mechanism may be related to the inhibition of inflammatory response, oxidative damage and pyroptosis.

## 1. Introduction

*Staphylococcus aureus* is an opportunistic pathogen, which is colonized in most healthy individuals, with infections ranging from mild skin infections to severe necrotizing pneumonia [1]. People with poor physical fitness, such as children and the elderly, are susceptible to *Staphylococcus aureus* pneumonia [2]. *Staphylococcus aureus* pneumonia has a rapid onset, and if not diagnosed and treated in a timely manner, it can lead to infectious death of patients. At present, antibiotic therapy is commonly used in clinic, especially vancomycin and linezolid are commonly used to treat infectious diseases caused by *Staphylococcus aureus* [3]. However, in recent years, reports on drug-resistant strains have been common. Due to the abuse of antibiotics, bacteria have different drug resistance mechanisms. These resistance mechanisms help bacteria evade the host immune system. All these lead to varying degrees of drug resistance of *Staphylococcus aureus*, resulting in poor clinical antibacterial effect [4,5]. Studies have shown that *methicillin-resistant Staphylococcus aureus* (MRSA) accounts for 20%-80% of *Staphylococcus aureus* infections, and has the highest mortality rate among all antibiotic-resistant pathogens [6]. MRSA is highly pathogenic because it can secrete a variety of virulence factors that can cause body damage, such as biofilms related α-hemolysin (Hla), staphylococcal protein A (SpA), staphylococcal sortase (Srt) [7–9], and leukocidin (PVL), lipoteichoic acid (LTA) and other pathogenic substances can also cause different severity of clinical manifestations [10,11]. Therefore, choosing a new treatment method to deal with *methicillin-resistant Staphylococcus aureus* pneumonia is an urgent medical problem that needs to be solved.

NOD-like receptor protein 3 (NLRP3) is an inflammasome that contains NLRP3, apoptosis-associated speck-like protein (ASC) and pro-caspase-1 to promote inflammatory responses [12]. Pathogens are common activators of NLRP3 [13], and bacterial RNA, exotoxins, and heme can stimulate the production of inflammatory vesicles [14,15]. Upon sensing noxious signals, NLRP3 activates caspase-1, which leads to the secretion of pro-interleukin 1β (IL-1β). Activated IL-1β is released out of cells and plays a critical role in lung inflammation [16]. Inhibition of NLRP3 and ASC activation, which in turn attenuates the onset of pyroptosis and inflammatory responses, may be a potential mechanism for the treatment of MRSA bacterial pneumonia.

Amygdalin is the main active pharmaceutical ingredient of bitter almond, which has broad-spectrum antibacterial, anti-inflammatory, antioxidant, and immune modulating effects [17]. It is also an important component of the Traditional Chinese Medicine compound, such as Lianhuaqingwen capsule, and Yinhuapinggan granule, to treat respiratory infection [18,19]. In our previous study, we found Yinhuapinggan granule had a good effect on the therapy of viral infectious respiratory disease, and amygdalin, as its main component played a crucial role [20]. So this study aims to further investigate the role of amygdalin in bacterial respiratory diseases. The commonly used antibiotics for *methicillin-resistant Staphylococcus aureus* pneumonia are mostly glycopeptide antibiotics, and vancomycin is generally the first choice [21], but the drug sensitivity of vancomycin is low, and the adverse reactions after use are large, so the effect of single use is not suboptimal. The researchers used vancomycin combined with levofloxacin for common treatment, and found that the levels of CRP and TNF-α were reduced after treatment. Levofloxacin can effectively bind to plasma proteins and improve the bioavailability of the drug [22]. At the same time, there are also many traditional Chinese medicine combined with levofloxacin to enhance the drug effect [23]. Because of the advantages of multi-component, multi-pathway and multi-target, traditional Chinese medicine can play an antibacterial and anti-inflammatory effect through multiple links, and bacteria are not easy to produce resistance to it [24]. To deal with antibiotic resistance and infectious diseases, traditional Chinese medicine can resist drug-resistant bacteria and their toxins, protect organs and tissues, regulate immune disorders, promote the repair of damaged tissues, and block microcirculation disorders. Traditional Chinese medicine can improve the clinical efficacy of anti-drug resistant bacterial infection, or use antibiotics to achieve the effect of "synergism and attenuation". The combination of traditional Chinese medicine and antibiotics is becoming a common clinical treatment [25,26].

Now the possible function and mechanisms of Amygdalin in pathogenic infection remain elusive, and whether NLRP3 pathway participates in Amygdalin regulating MRSA infection is not yet known. Based on this point of view, in this article, we studied the effects of Amygdalin based on the MRSA-induced human lung epithelial cells model and its potential mechanisms. At the same time, to explore whether amygdalin combined with levofloxacin has better effect.

## 2. Materials and methods

### 2.1. Reagents

Vancomycin (Batch Number: AF20050652) was purchased from Chengdu Alfa Biotechnology Co. Ltd. (Chengdu, China). Levofloxacin (Batch Number: H20020636) was purchased from Daiichi Sankyo Co. Ltd. (Beijing, China). Ampicillin (Batch Number: A100339-0005) / Cefalexin (Batch Number: A500280-0025)/ Gentamicin (Batch Number: A100210-0001) were purchased from Sangon Biotech Co. Ltd. (Shanghai, China). Vancomycin (Batch Number: D2127029) was purchased from Shanghai Aladdin Biochemical Technology Co. Ltd. (Shanghai, China). F12K cell culture medium was purchased from Boster Biological Technology Co. Ltd. (Wuhan, China). Fetal bovine serum was purchased from Thermo Scientific (Waltham, USA). Crystalline Violet Dyes and Hoechst 33342 were purchased from Beyotime Biotechnology Co. Ltd. (Shanghai, China). LDH test kit was purchased from Roche Biotechnology Co. Ltd. (Shanghai, China). Trizol was purchased from TaKaRa Biotechnology Co. Ltd. (Kusatsu, Japan). The RNA extraction reagent kit was purchased from Applied Biological Materials Inc. (Vancouver, Canada). Enzyme-linked immunosorbent assay (ELISA) reagent kits were purchased from Jiangsu Meibiao Biotechnology Co. Ltd. (Jiangsu, China).

### 2.2. Bacterial strain & cell strain

The clinical strains in this study were obtained from the clinical laboratory of the First People’s Hospital of Hangzhou, Zhejiang Province. A549 cell was obtained from the Cell Bank of Type Culture Collection of the Chinese Academy of Sciences (Shanghai, China).

### 2.3. Bacteria culture & minimal inhibitory concentrations (MIC) determination

MRSA were isolated from the Clinical Laboratory, Hangzhou First People’s Hospital. MRSA strains were inoculated in LB broth till mid-logarithmic phase under 200 rpm with 5% CO_2._ The bacterial solution was diluted in sterile saline to a concentration of 10^^5^ CFU/mL. MIC of Ampicillin (Amp), Cephalosporin cephalexin (Clx), Levofloxacin (Lev), Gentamycin (Gm), Vancomycin (Van) and Amygdalin (Am) solution for MRSA strain were determined by the broth micro-broth dilution method following the standards of the Clinical and Laboratory Standards Institute (CLSI) 2021-M100.

### 2.4. CCK-assay

A549 cells were cultured in F12K medium supplemented with 10% FBS and then placed at 37° C in an incubator containing 5% CO_2_. When the cells had grown to about 80% coverage, expanded cultures were performed. A549 cells in logarithmic growth phase were digested with trypsin, the cell density was adjusted to 10^^4^ cells/well, 100 μL per well was seeded in 96-well plate, and cultured overnight in cell incubator. The negative control group (without drug) and monomer administration groups with different concentrations were set up, each group had 6 multiple wells, and cultured for 24 h. 100 μL of a solution containing 10% CCK prepared with medium was added to each well, and the absorbance was measured at a wavelength of 450 nm using a microplate reader after 2 h of incubation.

### 2.5. LDH assay

A549 cells in logarithmic growth phase were harvested and digested with trypsin. The cell density was adjusted to 12×10^^4^ cells/well, and 500 μL per well was seeded in 24-well plates. The cells were cultured in a cell incubator overnight. Cells were infected with the optimal MOI while drugs were added and co-cultured to the indicated times. The supernatant was collected by centrifugation at 3000 rpm for 5 min. LDH kit was used to detect the protective effect of drugs on A549 cells infected with MRSA.

### 2.6. Hoechst staining

After cells were cultured overnight, monomeric compounds were added and infected at optimal MOI for the indicated times. The cells were washed three times with PBS to remove the supernatant and fixed with 4% paraformaldehyde for 20 min. After washing with PBS, Hoechst working solution was added and incubated at room temperature for 5 min. The working solution was removed by suction and washed 3 times with PBS before filming in the dark.

### 2.7. The anti-apoptotic effect measurement using flow cytometry

A549 cells in logarithmic growth phase were harvested and digested with trypsin. The cell density was adjusted to 12×10^^4^ cells/well, and 500 μL per well was seeded in 24-well plates. The cells were cultured overnight in an incubator. Bacterial solution at different MOI (number of bacteria: number of cells) was added to each well, cultured for the indicated time, and the supernatant was withdrawn to the centrifuge tube. After washing once with PBS, the supernatant was added to the centrifuge tube. Cells were digested with trypsin and added to a centrifuge tube and centrifuged at 1500 rpm for 5 min. The supernatant was discarded, washed again by PBS, and centrifuged. The supernatant was discarded, buffer was added, and after blowing evenly, the working solution was added. We used flow cytometry with Annexin-Ⅴ (annexin-V) and Propidium Iodide (PI) as detection signals to detect the phenomenon of apoptosis.

### 2.8. Adhesion assay and invasion assay

#### (1) Adhesion test

The cells were inoculated into 24-well cell culture plates and cultured for 24 h. The original cell culture medium was discarded, washed 3 times with PBS, and the monomer compounds were added at the optimal multiplicity of infection (MOI) for the indicated time. Each group was washed 3 times with PBS and soaked in 0.25% pancreatic enzyme100 μL and 0.5% TritonX-400 μL for 10 min. Then, 100 μL of the diluted solution was placed on LB medium and incubated at 37°C.

#### (2) Invasion test

Cells were infected using the same method as above. After washing three times with sterile PBS, 100 μL of basal cell culture medium containing 100 mg/mL vancomycin was added to each well for 40 min to kill extracellular bacteria. After washing three times with PBS and treated according to steps in the adhesion test.

### 2.9. Inflammatory cytokines measurement using ELISA

The cell supernatant was collected at the specified time point and centrifuged at 1000 rpm for 10 min. The levels of inflammatory factors such as IL-1β, TNF-α, IL-6, and IL-8 in the supernatant were detected by ELISA kit after the cell debris was discarded.

### 2.10. ROS measurement using flow cytometry

After cells were cultured overnight, monomeric compounds were added and infected at optimal MOI for the indicated times. The old medium was removed, washed once with PBS, and serum-free basal medium containing dye was added. The cells were incubated at 37°C for 20 min and the medium was removed. After washing three times with serum-free medium, digestion and centrifugation, cells were harvested. ROS formation was detected by flow cytometry.

### 2.11. The expression of genes was analyzed by qRT-PCR

0.1 mL of MRSA bacterial solution and 1 mL of LB liquid medium were added in each tube and incubated for 24 h. Bacterial total RNA was extracted using Trizol reagent, and then reverse transcribed to cDNA using a 5× All-In-One RT Mastermix. cDNA obtained by reverse transcription was used together with predesigned primers to detect the relative expression levels of *icaA*, *sarA*, *fnbb*, *hla*, *spA*, *blaZ*, *mecA* using qRT- PCR. All primers were designed and purchased from Shanghai Sangon Biotech. S16 was used as an internal control for comparison and the results were calculated using the ΔΔCt method. After extraction of total RNA from cell by Trizol reagent, the rest of the procedure was the same as above. GAPDH was used as an internal control for comparison and the results were calculated using the ΔΔCt method. The primer sequences were shown in Table 1.

**Table 1:** Primer sequences for qRT-PCR.

Gene	Sequence (5’-3’) forward	Sequence (5’-3’) backward
icaA	ACACTTGCTGGCGCAGTCAA	TCTGGAACCAACATCCAACA
sarA	GGCAAATGTATCGAGCAAGATG	GCTTCAGTGATTCATTTATTTACTC
fnbb	CGTTATTTGTAGTTGTTTGTGTT	TGGAATGGGACAAGAAAAAGAA
hla	GAATCCTGTCGCTAATGCC	TGATTGCCATATACCGGGTT
spA	GCGCAACACGATGAAGCTCAACAA	ACGTTAGCACTTTGGCTTGGATCA
blaZ	GCTTTAAAAGAACTTATTGAGGCTTC	CCACCGATYTCKTTTATAATTT
mecA	GGTGGCTGGGGGGTAGATGTATTAACTGG	GCTTCTTTTGAAATACATGGTATTTTTCGATC
16S	GCTGCCCTTTGTATTGTC	AGATGTTGGGTTAAGTCCC
NLRP3	TCAGTCCCACACACAGCAAT	CATCAATGCTGCTTCGACAT
ASC	CTCCAGGTCCATCACCAAGT	AGACCACCAGCCAAGACAAG
IL-1β	AGCTTCTCCACAGCCACAAT	GCTGCTTCCAAACCTTTGAC
GAPDH	GAGTTGCTGTTGAAGTCGCA	TGGCCTTCCGTGTTCCTAC

### 2.12. Statistical analysis

Results were analyzed using GraphPad Prism 8.0 (GraphPadSoftware, USA) and expressed as mean ± standard deviation (SD). One-way analysis of variance (ANOVA) was used for statistical significance analysis. Comparisons between more than two groups were performed using the *Tukey’s* test. Comparisons between two groups were performed using the *student’s* t-test. P < 0.05 was regarded as statistically significant in the analyses.

## 3. Results

### 3.1. Bacterial growth curves and drug resistance assays

**Fig 1:**
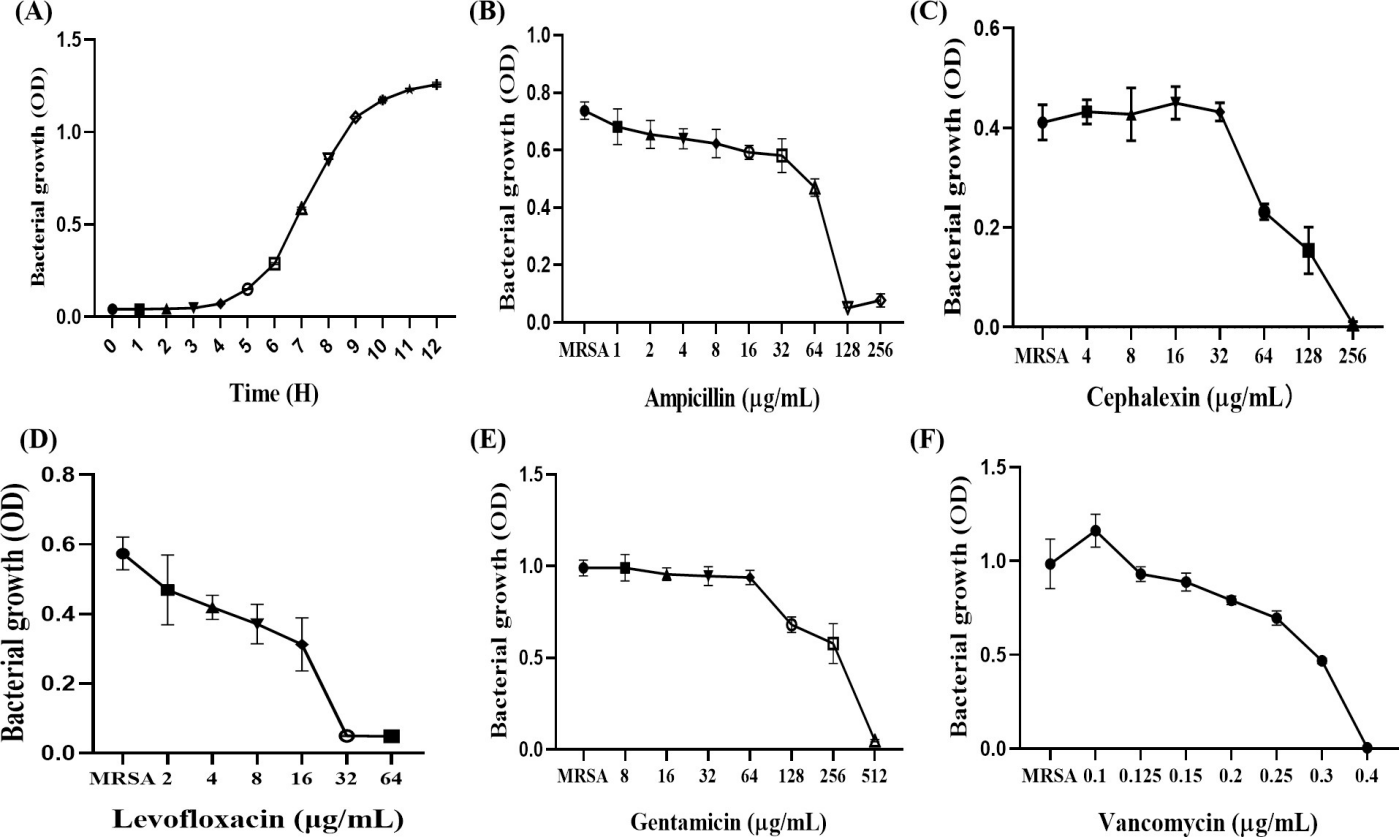
Bacterial growth curves and drug resistance assays. (A)MRSA growth curve. (B) MIC of Ampicillin for MRSA. (C) MIC of Cefalexin for MRSA. (D) MIC of Levofloxacin for MRSA. (E) MIC of Gentamicin for MRSA. (F) MIC of Vancomycin for MRSA.

The bacterium reached the logarithmic growth phase at 6 h and entered the plateau phase after 11 h (Fig 1A). Drug resistance test showed that the bacteria belonged to multi-drug resistant bacteria, which was resistant to β-lactam antibiotics ampicillin, cephalosporin cephalexin, quinolone antibiotics levofloxacin, aminoglycoside antibiotics gentamicin, but not to glycopeptide antibiotics vancomycin (Fig 1B–1F).

### 3.2. The antibacterial effect of amygdalin

**Fig 2:**
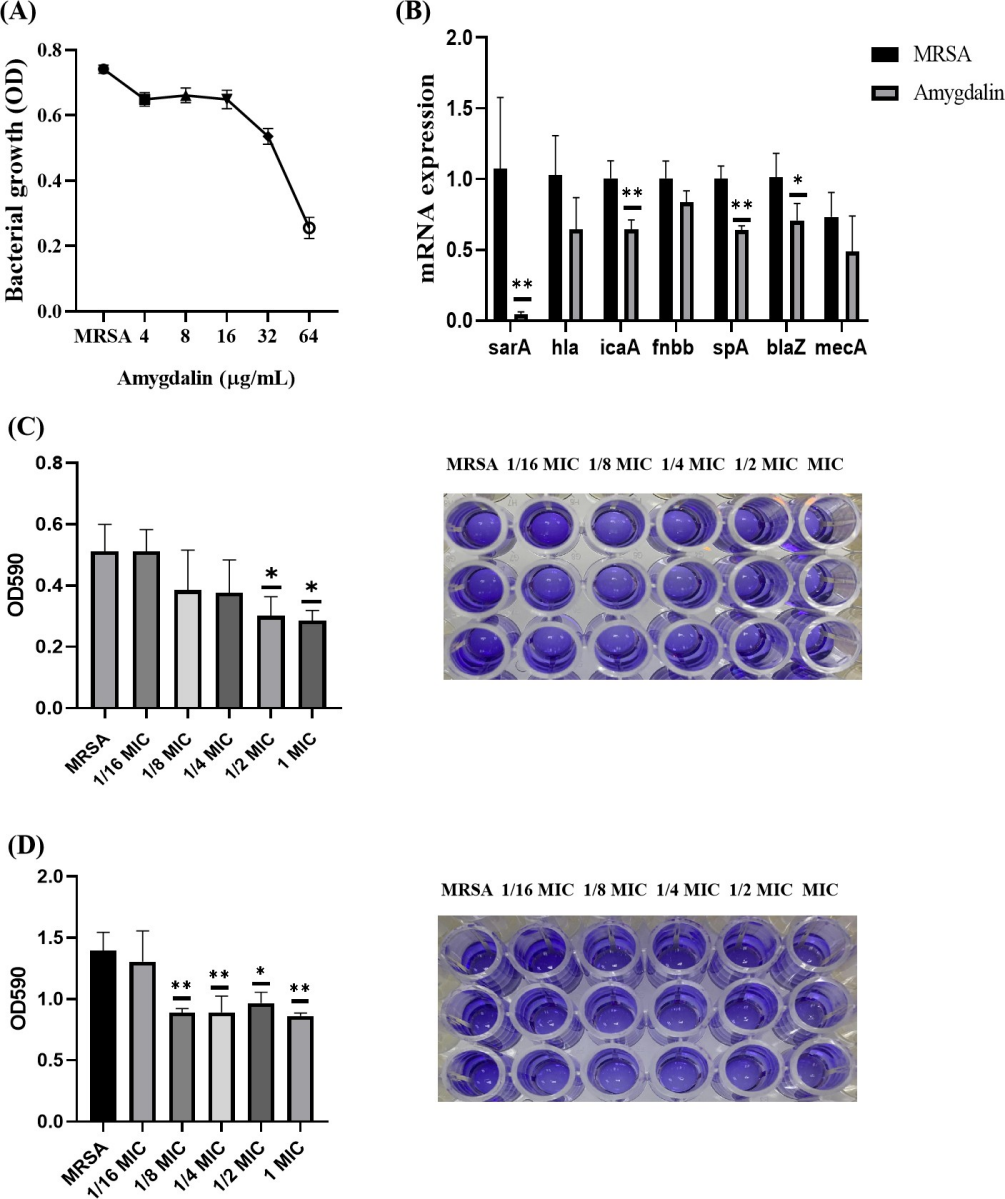
Effect of amygdalin on MRSA growth and biofilm and regulation of related gene expression. (A) MIC of Amygdalin for MRSA. (B) The mRNA levels of *sarA*、*hla*、*icaA*、*fnbb*、*spA*、*blaZ*、*mecA*. (C) Amygdalin inhibits MRSA biofilm formation. (D) Amygdalin clears biofilms formed by MRSA. Results are representative of at least three experiments; error bars indicate SD; *p < 0.05, **p < 0.01 compared with control. (This work is licensed under Creative Commons Attribution 4.0 International. To view a copy of this license, visit https://creativecommons.org/licenses/by/4.0/).

Microwell dilution method was used to detect the effect of amygdalin on MRSA. After 20 h of bacterial culture, the culture medium was clear without turbidity when the concentration of amygdalin was 64 μg/mL, and the MIC value of amygdalin to MRSA was 64 μg/mL (Fig 2A). For the detection of MRSA drug resistance genes, after amygdalin treatment of MRSA, qRT-PCR results showed that amygdalin could down-regulate the expression of biofilm related *sarA*, *fnbb* and *icaA* genes. Amygdalin also significantly down-regulated the virulence-related *hla* and *spA* genes, but only *spA* showed significant difference compared with MRSA. Amygdalin also significantly down-regulated the expression of *blaZ* gene. Amygdalin down-regulated the expression of *mecA*, but there was no significant difference compared with MRSA (Fig 2B).

Biofilms were stained with crystal violet to study the effect of amygdalin on biofilm proliferation and clearance. The results showed that amygdalin at the concentration of 1/2 MIC has a significant inhibitory effect on the growth of biofilm compared with MRSA group, and the inhibitory effect was more obvious with the increase of the concentration (Fig 2C). The results of crystal violet staining of amygdalin at 1/8 MIC were significantly different from those of the MRSA group, indicating that the resultant biofilm could be removed in a concentration-dependent manner. Amygdalin may also play an antibacterial role by inhibiting and eliminating biofilm formation (Fig 2D).

### 3.3. Protective effect of amygdalin and levofloxacin in combination

**Fig 3:**
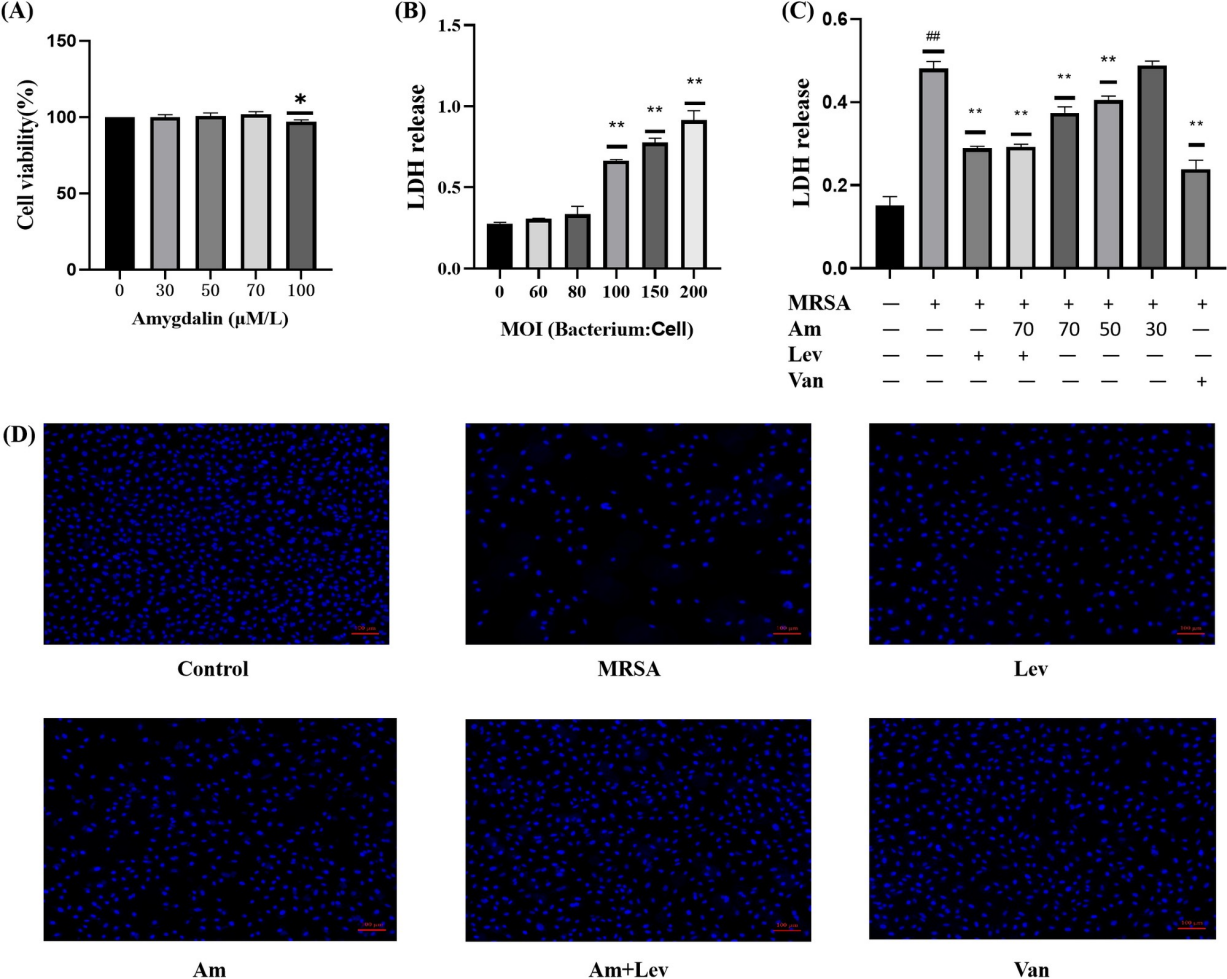
Protective effect of amygdalin and levofloxacin in combination. (A) CCK-assay analyzed the cell viability after 24 h incubation with various concentrations of Am. (B) Optimal concentration and time of infection. (C) Protective effect of amygdalin combined with levofloxacin on MRSA infected cells. (D) Hoechst fluorescent staining. Results are representative of at least three experiments; error bars indicate SD; ^#^*p* < 0.05, ^##^*p* < 0.01 compared with control. *p < 0.05, **p < 0.01 compared with MRSA.

A549 cells were treated with different concentrations of amygdalin for 24 h. CCK- assay was used to detect the survival rate of A549 cells. The maximum safe concentration of amygdalin was 70 μM/L, and 70, 50, and 30 μM/L were used as experimental concentrations in subsequent experiments (Fig 3A). An in *vitro* model of pneumonia was established by infecting A549 cells with MRSA. MRSA at MOI 60, 80, 100, 150 and 200 were used to stimulate A549 cells for 9 h, respectively. The results of LDH showed that with the increase of MOI, the release of LDH increased, and the damage of cells was gradually aggravated. When MOI was equal to 100, the LDH release was significantly increased compared with the control group (Fig 3B). Therefore, we selected MOI 100 infection for 9 h as the condition for subsequent experiments. MRSA was selected as a multi-drug resistant strain, which was resistant to the quinolone levofloxacin. The study hopes to further test whether the combination of levofloxacin and amygdalin will be more effective, and at the same time, vancomycin is selected as a positive drug for comparison. The results showed that different doses of amygdalin could reduce the release of LDH in the infected cells, and levofloxacin could also reduce the release of LDH, but the combination of high dose of amygdalin and levofloxacin has no better effect (Fig 3C). The results of Hoechst staining showed that amygdalin could protect the cells from injury, and amygdalin combined with levofloxacin had better effect than amygdalin alone (Fig 3D).

### 3.4. Amygdalin reduces cell apoptosis caused by MRSA infection

**Fig 4:**
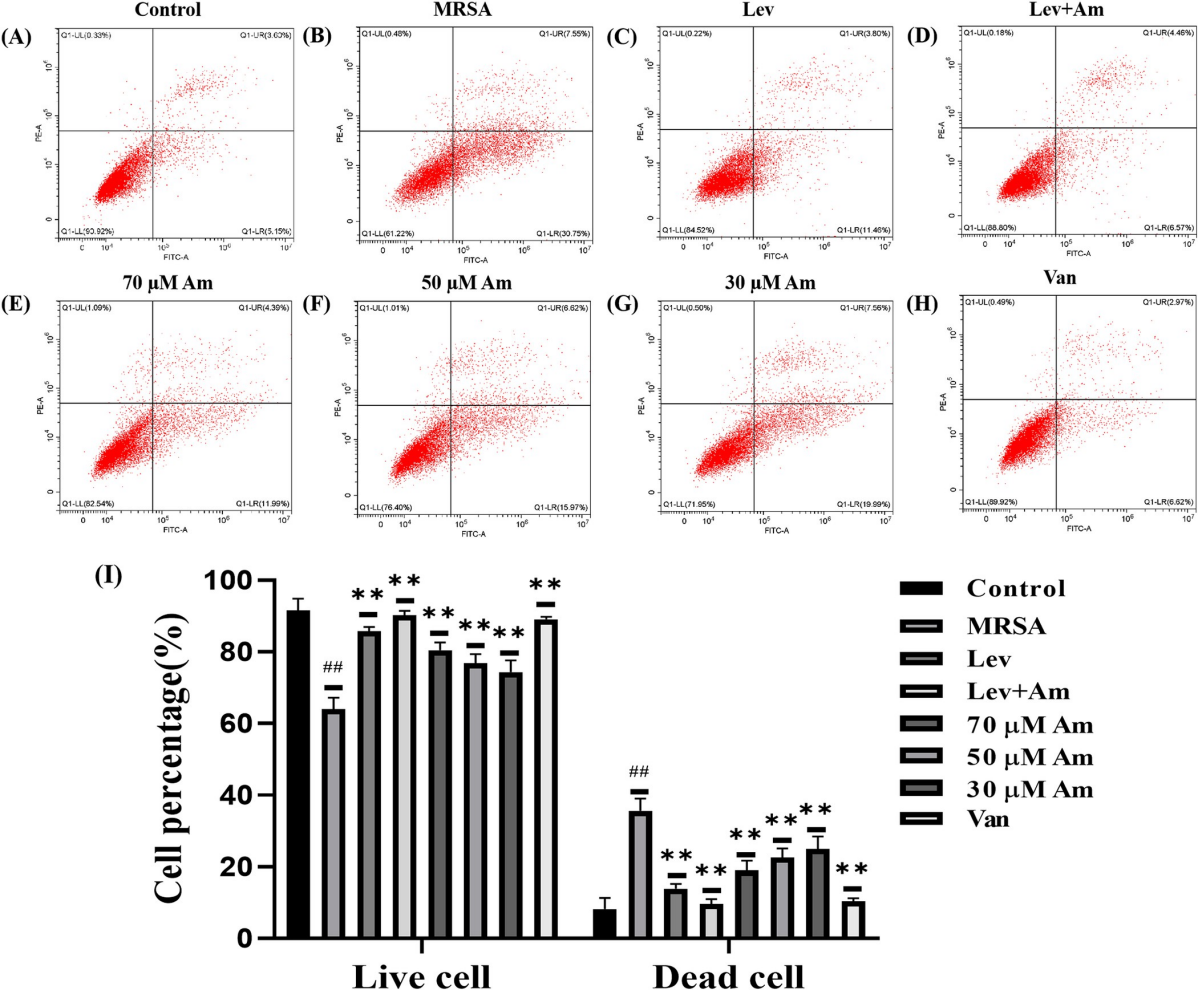
Flow cytometry was used to detect the protective effect of amygdalin on infected cells. (A) control group. (B) MRSA group. (C) Levofloxacin group. (D) Amygdalin combined with levofloxacin group. (E) Amygdalin high-dose group. (F) Amygdalin medium-dose group. (G) Amygdalin low-dose group. (H) Vancomycin group. (I) Flow cytometry was used to identify apoptosis.

We used Annexin V-FITC for the assay to investigate whether amygdalin protects cells from MRSA-induced damage. The results showed that compared with the control group (Fig 4A), the proportion of dead cells was significantly increased and the cell damage was obvious after MRSA infection (Fig 4B). Amygdalin reduced the proportion of MRSA-induced dead cells and increased the proportion of living cells, with a concentration gradient effect (Fig 4E–4G). Levofloxacin also provided protection against apoptotic cells (Fig 4C). Amygdalin in combination with levofloxacin is better than single drug protection (Fig 4D).

### 3.5. Effect of amygdalin on the adhesion and invasion ability of MRSA

**Fig 5:**
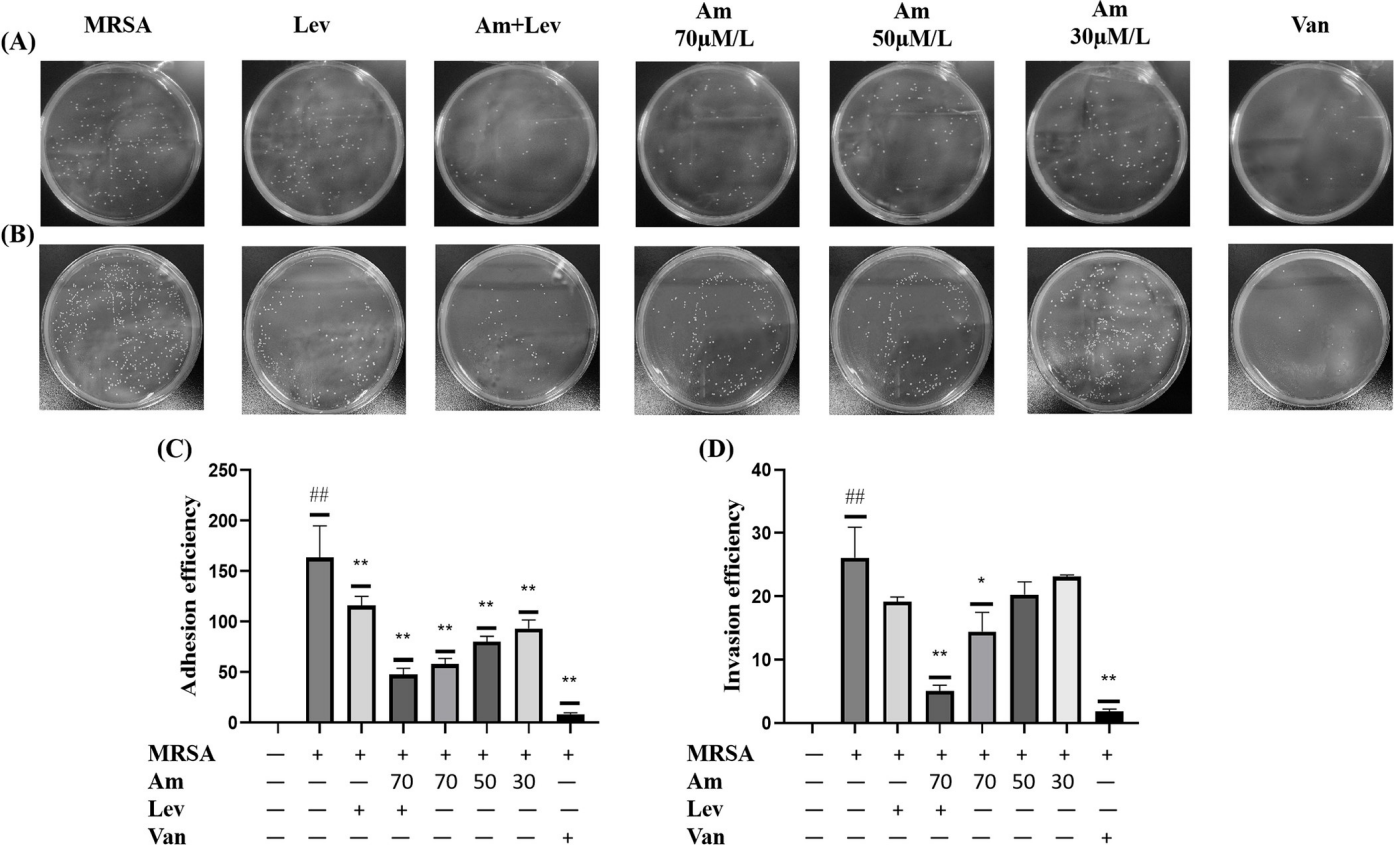
Effect of amygdalin on the adhesion and invasion ability of MRSA. (A) Coating was used to detect MRSA adhesion. (B) Coating was used to detect MRSA invasion. (C) Adhesion efficiency. (D) Invasion efficiency. Results are representative of at least three experiments; error bars indicate SD; ^#^*p* < 0.05, ^##^*p* < 0.01 compared with control. *p < 0.05, **p < 0.01 compared with MRSA. (This work is licensed under Creative Commons Attribution 4.0 International. To view a copy of this license, visit https://creativecommons.org/licenses/by/4.0/).

Adhesion assay showed that amygdalin could reduce the adhesion of MRSA to A549 cells, and the adhesion efficiency of levofloxacin was significantly reduced compared with that of the model group. After high, medium and low doses of amygdalin administration, the adhesion of MRSA was significantly reduced compared with that of the model group, and showed a concentration gradient effect. After the combination of levofloxacin and amygdalin in the high-dose group, there was no significant difference in the adhesion efficiency of MRSA compared with the high-dose amygdalin group, so the combination group did not inhibit the adhesion of MRSA to A549 cells more than the single administration group (Fig 5A and 5C). The invasion assay showed that amygdalin could reduce the invasion effect of MRSA on A549 cells. Levofloxacin reduced the invasion efficiency compared with the model group, but there was no significant difference. After high, medium and low doses of amygdalin administration, only the high-dose amygdalin group had a significant difference compared with the model group. The ability to inhibit MRSA invasion was stronger, indicating that the combined drug administration group had a stronger ability to inhibit MRSA invasion of A549 cells than the single drug administration group (Fig 5B and 5D).

### 3.6. Effect of amygdalin on inflammatory markers in A549 cells infected with MRSA

**Fig 6:**
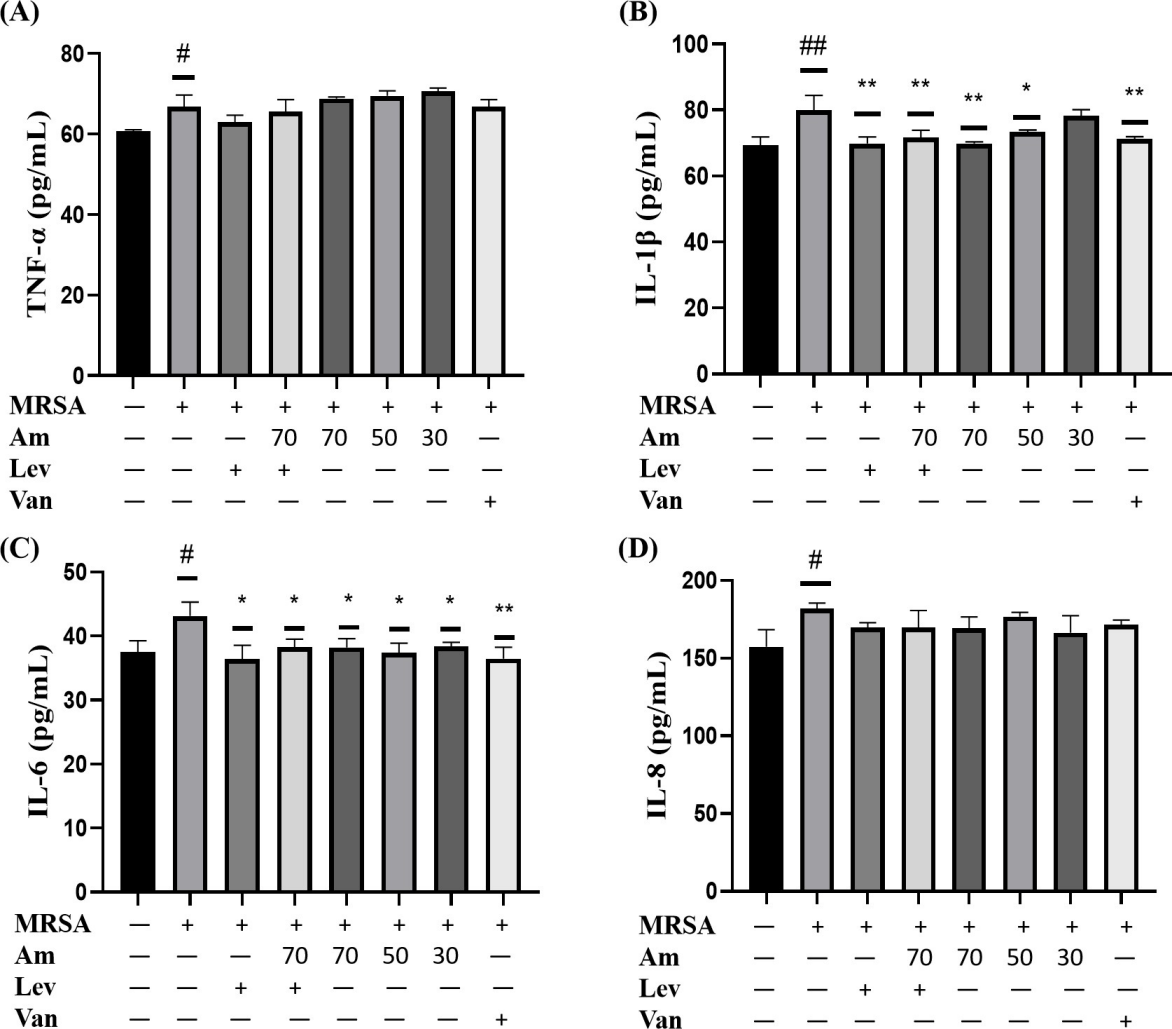
Effect of amygdalin on cellular inflammatory response induced by MRSA. (A) TNF-α expression in the cell supernatant (B) IL-1β expression in the cell supernatant (C) IL-6 expression in the cell supernatant. (D) IL-8 expression in the cell supernatant. Results are representative of at least three experiments; error bars indicate SD; ^#^*p* < 0.05, ^##^*p* < 0.01 compared with control. *p < 0.05, **p < 0.01 compared with MRSA.

As shown in Fig 6A, compared with the control group, the expression of TNF-α in the cell supernatant of MRSA infection group was significantly increased. After the administration of levofloxacin and amygdalin, the results showed that levofloxacin could reduce the expression of TNF-α, but there was no significant difference with the control group. The combination group was also less effective than the single drug group (Fig 6A). Compared with the control group, the expression of IL-1β in the cell supernatant of the MRSA infection group was significantly increased. After the administration of levofloxacin and amygdalin, the results showed that levofloxacin could significantly reduce the expression of IL-1β, and high and medium doses of amygdalin could significantly reduce the expression of IL-1β (Fig 6B). Compared with the control group, the expression of IL-6 in the cell supernatant of MRSA infection group was significantly increased. After the administration of levofloxacin and amygdalin, the results showed that levofloxacin could significantly reduce the expression of IL-6, and high, medium and low doses of amygdalin could significantly reduce the expression of IL-6, and the effect of combination group was not as good as that of single administration group (Fig 6C). Compared with the control group, the expression of IL-8 in the cell supernatant of MRSA infection group was significantly increased, and the results were basically the same as TNF-α expression. Levofloxacin could reduce the expression of IL-8, but there was no significant difference between the control group and levofloxacin. The effect of the combination group was almost the same as that of the single administration group (Fig 6D).

### 3.7. Effect of amygdalin on ROS production in A549 cells infected with MRSA

**Fig 7:**
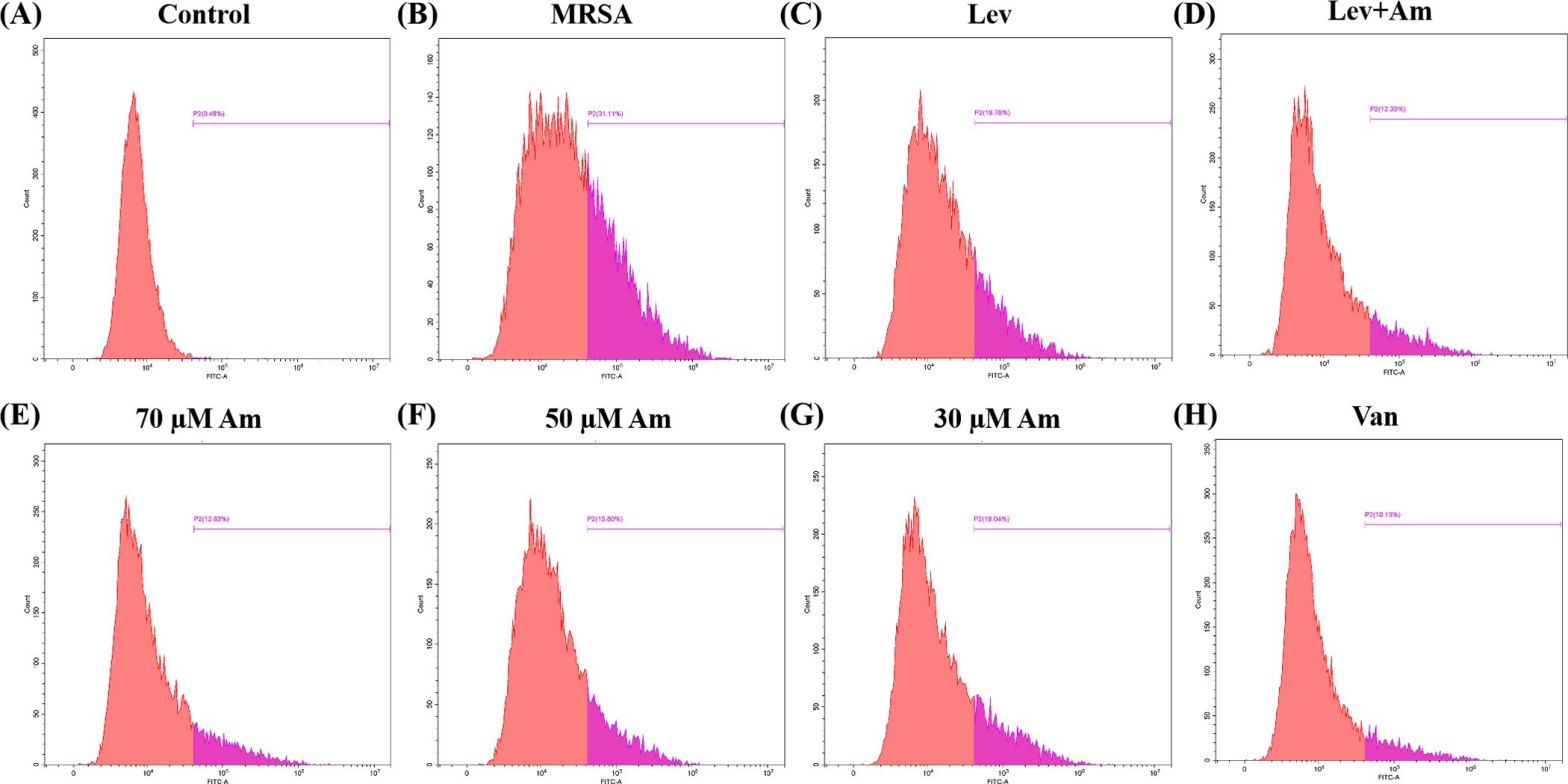
Effect of amygdalin on ROS production in A549 cells infected with MRSA. (A) Control group. (B) MRSA group. (C) Levofloxacin group. (D) Amygdalin combined with levofloxacin group. (E) Amygdalin high-dose group. (F) Amygdalin medium-dose group. (G) Amygdalin low-dose group. (H) Vancomycin group.

To verify whether amygdalin could regulate the ROS expression caused by MRSA infection, we probed ROS concentrations using flow cytometry. Flow cytometry results showed the expression of ROS was significantly higher in the MRSA-infected group compared with the control group (Fig 7A and 7B). ROS expression levels were significantly reduced after levofloxacin administration (Fig 7C). Amygdalin treatment also significantly reduced the expression of ROS (Fig 7E–7G). There was no significant difference in ROS expression between the amygdalin combined levofloxacin treatment group (Fig 7D) and the high-dose amygdalin treatment group (Fig 7E). Therefore, amygdalin can protect cells from the damage of ROS produced by MRSA.

### 3.8. Effect of amygdalin on pyroptosis pathway in A549 cells infected with MRSA

**Fig 8:**
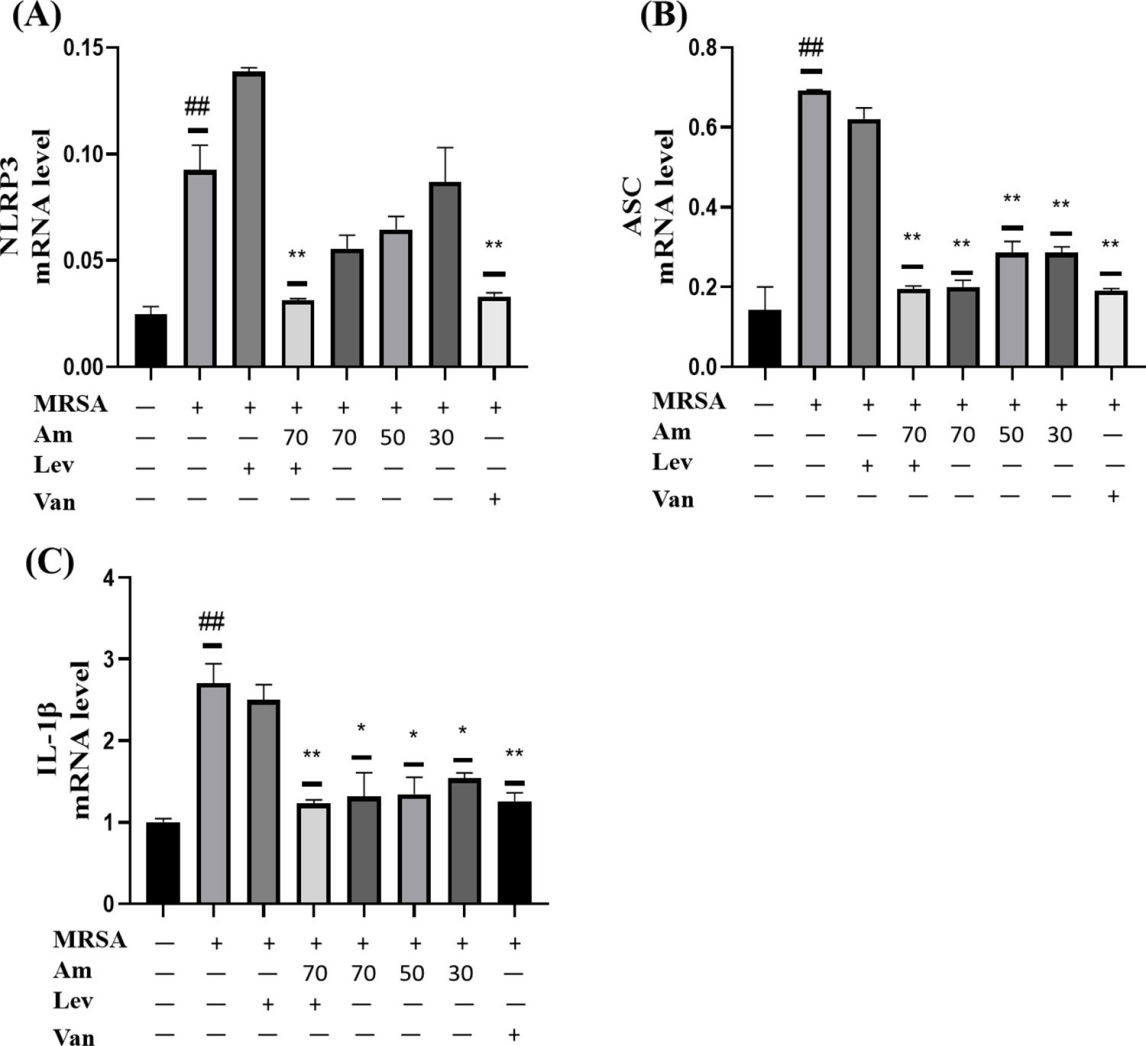
Effect of amygdalin on pyroptosis pathway in A549 cells infected with MRSA. (A) *NLRP3* mRNA expression. (B) *ASC* mRNA expression. (C) *IL-1β* mRNA expression. Results are representative of at least three experiments; error bars indicate SD; ^#^*p* < 0.05, ^##^*p* < 0.01 compared with control. *p < 0.05, **p < 0.01 compared with MRSA.

MRSA can cause pyroptosis after infecting cells. Therefore, we further investigated the effect of amygdalin on the NLRP3/ASC/IL-1β pyroptosis signaling pathway in MRSA infected cells. The results of qRT-PCR showed that the expression of *NLRP3* mRNA in MRSA infected group was significantly higher than that in the control group. After levofloxacin administration, the expression of *NLRP3* mRNA increased. After the administration of different doses of amygdalin, the expression of *NLRP3* mRNA was down regulated, but there was no significant difference compared with the model group, but after the combination of high doses of amygdalin and levofloxacin, the expression of *NLRP3* mRNA was significantly down regulated compared with the model group. (Fig 8A). The expression of *ASC* mRNA in MRSA infected group was significantly higher than that in the control group. *ASC* mRNA expression was down regulated after levofloxacin administration, but the difference was not statistically significant. After the administration of different doses of amygdalin, the expression of *ASC* mRNA was significantly down regulated compared with the model group. After the administration of levofloxacin combined with high-dose amygdalin, the expression of *ASC* mRNA was basically the same as that of the control group (Fig 8B). After MRSA infection, the expression of *IL-1β* mRNA in the infected group was significantly higher than that in the control group. After levofloxacin administration, the expression of *IL-1β* mRNA was down regulated, but there was no significant difference. After different doses of amygdalin administration, the expression of *IL-1β* mRNA was significantly down regulated, and the effect of levofloxacin combined with high-dose amygdalin on the expression of *IL-1β* mRNA was basically the same as that in the positive drug group (Fig 8C).

## 4. Discussion

MRSA pneumonia induced by injury is the important pathologic basis of pneumonia, reduce the resistance of MRSA and alleviate lung epithelial cell damage is a potential therapeutic target. Amygdalin is the main active ingredient of bitter almond, which has antibacterial, anti-inflammatory and antioxidant properties [27–29]. Our study confirmed that amygdalin had a promising antibacterial effect in *vitro*. However, its antibacterial mechanism is still unclear. The drug resistance of MRSA is mainly related to biofilm and drug resistance genes. The most prominent characteristics of MRSA biofilm are its strong adhesion and strong drug resistance, which enable the bacteria to resist the host immune response and escape the killing of antibiotics [30]. *Staphylococcus aureus* co-regulator A (sarA), as a global virulence regulatory system of *Staphylococcus aureus*, can control a variety of adhesion proteins to regulate intercellular adhesion of *Staphylococcus aureus*, such as fibronectin binding protein (fnbb) and intercellular adhesion gene (*icaA*), and play an active role in biofilm formation [31,32]. Therefore, some studies have reduced the virulence of MRSA by inhibiting the expression of *sarA* [33]. Our study also showed that amygdalin inhibited biofilm growth and formation. Moreover, amygdalin significantly reduced the expression of biofilm related genes *sarA*, *icaA* and *fnbb*. Studies have demonstrated that amygdalin can act as an antimicrobial agent by affecting biofilms. Also MRSA resistance is associated with the resistance genes *blaZ* and *mecA*. They produce β-lactamase and penicillin-binding protein 2a(PBP2a), respectively, induced by β-lactam antibiotics [34]. Amygdalin can down-regulate the expression of the drug resistance genes *blaZ* and *mecA*. These results indicate that amygdalin can affect the expression of β-lactamase through *blaZ* gene and reduce the drug resistance of MRSA. Staphylococcal protein A (SpA) and α-haemolysin (hla) are secreted by MRSA as virulence factors that can cause damage to the organism. SpA will make MRSA to escape the body’s immune response, and at the same time can cause a B cell apoptosis [35]. Hla can induce the inflammasome NLRP3 in the target cells to cause the body to produce an inflammatory response. Studies have found that amygdalin can play an antibacterial and anti-inflammatory role by reducing the expression of virulence factors [36].

Following bacterial infection, the host can respond to pathogen infection in several ways, such as activating apoptosis-related cells and inducing inflammatory responses [37]. Studies have shown that MRSA infection cause lung epithelial cell apoptosis after enhancement [38]. And amygdalin reduces cell death and protects against cell damage in lung epithelial cells [27]. Our study found that whenever MRSA infected A549 cells, cell viability decreased and the number of apoptotic cells increased. However, bitter amygdalin treatment resulted in a significant increase in cell viability and a decrease in the number of apoptotic cells. These results indicate that amygdalin has a protective effect on A549 cells infected with MRSA, and it has a better protective effect when combined with antibiotics. MRSA infection requires attachment to host cells [39]. And MRSA has the ability of invasion of non-macrophages, and can result in persistent infection, eventually lead to a host cell death [40]. Invasion of cells by MRSA will cause the bacteria to evade the host’s immune system, reducing their effectiveness against certain antibiotics, which is one of the reasons for the development of bacterial resistance [41]. For the adhesion and invasion ability of MRSA, amygdalin effectively reduced the adhesion and invasion of MRSA to A549 cells.

Pneumonia caused by *staphylococcus aureus* induces pathophysiologic changes including oxidative stress and inflammation, which aggravate the body damage [5,42]. *Staphylococcus aureus* infection causes abnormal mitochondrial energy production, Ca^2+^ overload, and excessive ROS production. Excessive ROS can destroy the cellular proteins, lipids and DNA, causing cell death and apoptosis, oxidative stress. It also upregulates the expression of inflammatory cytokines such as IL-1β and TNF-α, which leads to cellular tissue damage in the body [43,44]. When NLRP3 is activated by stimuli such as *Staphylococcus aureus* and α-hemolysin, it promotes the activation and maturation of ASC, which in turn regulates and promotes the maturation of pro-inflammatory cytokines IL-1β and IL-6, leading to increased cellular inflammatory damage [45]. To further understand the mechanism of amygdalin in protecting A549 cells against MRSA infection. We performed anti-inflammatory, anti-oxidation, and anti-pyroptosis assays. Amygdalin was found to reduce the expression of IL-1β and IL-6 in the supernatant of A549 cells. However, there was no synergistic effect in the group of antibiotics in combination with amygdalin. As for the ROS generated by oxidative stress, the flow results showed that amygdalin could reduce the expression of ROS in A549 cells after MRSA infection and have the effect of antioxidant. For cellular inflammasome genes, amygdalin could down-regulate the expression of *NLRP3/ASC/IL-1β* gene targets, which are important in the pyroptosis pathway. These results indicated that amygdalin could protect MRSA-infected A549 cells from pyroptosis damage.

## 5. Conclusion

Amygdalin shows good antibacterial activity in *vitro* and has an effect on the drug resistance mechanism of MRSA. Amygdalin also has a protective effect on A549 cells infected with MRSA, and the effect is better when combined with levofloxacin. It is also proved that amygdalin can reduce the adhesion and invasion of MRSA to A549 cells. Amygdalin combined with levofloxacin has a better effect on the invasion of MRSA than a single drug. Amygdalin also has anti-inflammatory effect, which can significantly reduce the increase of IL-1β and IL-6 inflammatory factors caused by infection. For the antioxidant studies, amygdalin reduced the elevation of ROS expression caused by infection. The protective mechanism of amygdalin on A549 cells may be related to the inhibition of NLRP3, ASC and IL-1β pyroptosis pathway expression. It is hoped that these findings will help and support the use of antimicrobial agents and new treatment modalities.

## Supporting information

S1 Fig MRSA infected A549 cells for 6h. (TIF)

S2 Fig MRSA infected A549 cells for 12h. (TIF)

S1 File Raw data files for bacterial growth curves and drug resistance analysis. (RAR)

S2 File Raw data files for effect of amygdalin on MRSA growth and biofilm and regulation of related gene expression. (RAR)

S3 File Raw data files for protective effect of amygdalin and levofloxacin in combination. (RAR)

S4 File Raw data files for flow cytometry detect the protective effect of amygdalin on infected cells. (RAR)

S5 File Raw data files for effect of amygdalin on the adhesion and invasion ability of MRSA. (RAR)

S6 File Raw data files for effect of amygdalin on cellular inflammatory response induced by MRSA. (RAR)

S7 File Raw data files for effect of amygdalin on ROS production in A549 cells infected with MRSA. (RAR)

S8 File Raw data files for effect of amygdalin on pyroptosis pathway in A549 cells infected with MRSA. (RAR)

S9 File Raw data files for MRSA infected A549 cells for 6h. (RAR)

S10 File Raw data files for MRSA infected A549 cells for 12h. (RAR)
