# Supplementary figures and images for: Amygdalin prevents multidrug-resistant Staphylococcus aureus-induced lung epithelial cell injury by regulating inflammation and oxidative stress
Source: PLoS One. 2024 Sep 16;19(9):e0310253. doi: 10.1371/journal.pone.0310253 (PMC11404817; doi:10.1371/journal.pone.0310253)

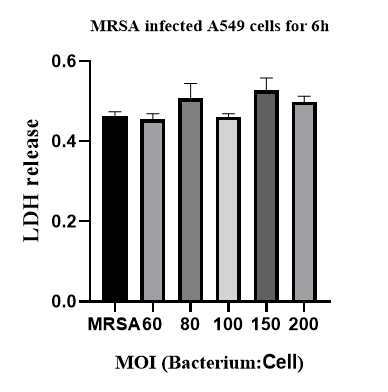

Supplement: S1 Fig — (TIF) [file pone.0310253.s001.tif]

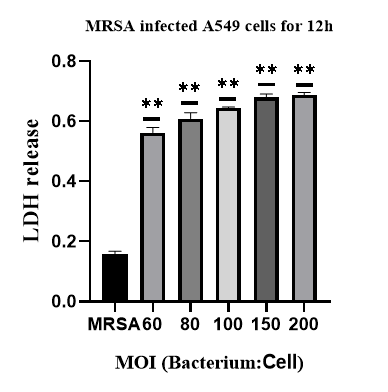

Supplement: S2 Fig — (TIF) [file pone.0310253.s002.tif]
